# Trophic Cascades Induced by Lobster Fishing Are Not Ubiquitous in Southern California Kelp Forests

**DOI:** 10.1371/journal.pone.0049396

**Published:** 2012-11-29

**Authors:** Carla M. Guenther, Hunter S. Lenihan, Laura E. Grant, David Lopez-Carr, Daniel C. Reed

**Affiliations:** 1 Interdepartmental Graduate Program in Marine Science, University of California Santa Barbara, Santa Barbara, California, United States of America; 2 Bren School of Environmental Science and Management, University of California Santa Barbara, Santa Barbara, California, United States of America; 3 Department of Geography, University of California Santa Barbara, Santa Barbara, California, United States of America; 4 Marine Science Institute, University of California Santa Barbara, Santa Barbara, California, United States of America; The Australian National University, Australia

## Abstract

Fishing can trigger trophic cascades that alter community structure and dynamics and thus modify ecosystem attributes. We combined ecological data of sea urchin and macroalgal abundance with fishery data of spiny lobster (*Panulirus interruptus*) landings to evaluate whether: (1) patterns in the abundance and biomass among lobster (predator), sea urchins (grazer), and macroalgae (primary producer) in giant kelp forest communities indicated the presence of top-down control on urchins and macroalgae, and (2) lobster fishing triggers a trophic cascade leading to increased sea urchin densities and decreased macroalgal biomass. Eight years of data from eight rocky subtidal reefs known to support giant kelp forests near Santa Barbara, CA, USA, were analyzed in three-tiered least-squares regression models to evaluate the relationships between: (1) lobster abundance and sea urchin density, and (2) sea urchin density and macroalgal biomass. The models included reef physical structure and water depth. Results revealed a trend towards decreasing urchin density with increasing lobster abundance but little evidence that urchins control the biomass of macroalgae. Urchin density was highly correlated with habitat structure, although not water depth. To evaluate whether fishing triggered a trophic cascade we pooled data across all treatments to examine the extent to which sea urchin density and macroalgal biomass were related to the intensity of lobster fishing (as indicated by the density of traps pulled). We found that, with one exception, sea urchins remained more abundant at heavily fished sites, supporting the idea that fishing for lobsters releases top-down control on urchin grazers. Macroalgal biomass, however, was positively correlated with lobster fishing intensity, which contradicts the trophic cascade model. Collectively, our results suggest that factors other than urchin grazing play a major role in controlling macroalgal biomass in southern California kelp forests, and that lobster fishing does not always catalyze a top-down trophic cascade.

## Introduction

Trophic cascades, in which predator-prey interactions control the composition and structure of ecological communities across two or more trophic levels in a food web have been reported in terrestrial, aquatic, and marine ecosystems [1], [2]. In a top-down cascade, changes in the abundances of predators act to alter the abundances of grazers, which in turn affect the biomass of primary producers [3]. The degree to which predators indirectly influence primary producers depends upon biotic and abiotic conditions that vary in space and time in response to physical disturbance, the availability of resources to primary producers, and the behavior of individual consumers [4]. As such, our understanding of how and why trophic cascades vary spatially and temporally is far from complete, which limits our ability to successfully manage and protect natural ecosystems in the face of increasing threats from anthropogenic disturbances and socio-economic pressures.

In coastal marine ecosystems top-down trophic cascades have been linked to the removal of top predators through fishing [5]–[12]. Frequently cited examples of marine trophic cascades come from kelp forests, in which top predators, such as sea otters [10], [11], fishes [6], [7], [12], and lobsters [5], [7], [13]–[15], are reduced in abundance by humans, leading to a relaxation in top-down control on sea urchin grazers and a decline in macroalgal abundance due to enhanced herbivory. The trophic cascade triggered by fishing in kelp forests includes a fourth trophic level occupied by humans, and depends on strong top-down interactions involving: (1) humans capturing predators of sea urchins (e.g., lobsters, fishes, and sea otters), (2) predators consuming urchins, and (3) urchins grazing macroalgae. The importance of trophic cascades as the primary determinant of community structure in kelp forest systems has been challenged because macroalgal abundance can vary greatly across space and time for many reasons other than grazing intensity [16], [17]. Therefore, the underlying cascade involving fishing, lobsters, urchins, and macroalgae may not be ubiquitous.

Weak top-down control implies that macroalgal abundance is unrelated to the abundance of urchins and their predators, and to fishing pressure on them. Nutrient availability, wave disturbance, sedimentation, and interactions among these factors are widely recognized as other drivers of macroalgal population dynamics [18], [19]. When nutrient supply is sufficiently high, kelp production can overwhelm the capacity of grazers to control kelp abundance [20]. Populations of grazers can similarly be affected by factors other than predation and fishing, as recruitment variability [21], [22], disease [23], storm disturbance [24], and hydrodynamic conditions [25], [26] have all been shown to influence the local abundance of sea urchins. Larger-scale processes such as El Niño-Southern Oscillation events (ENSO) can have regional effects that permeate throughout the food web by altering species abundances and the interactions among species in different trophic levels [27].

Correlative evidence for the cascading effects of fishing in marine ecosystems [5], [28], [29] has fueled calls for more intensive conservation, including the establishment of marine protected areas that prohibit fishing [7], [30], [31]. Most studies examining the effects of marine reserves have shown increased biomass and diversity in no-fishing areas compared with fished areas, which has further validated pleas for increased conservation [30]. The vast majority of this work, although highly informative, did not explore the direct effects of fishing intensity on trophic cascades, but rather assumed that spatial variation in predator and grazer abundance, and therefore predation and grazing intensity, was due to the presence versus absence of fishing [23], [26], [32]. Assuming that comparisons of predator density inside versus outside of reserves provide a good estimate of fishing impacts can be problematic because unprotected areas often have large differences in fishing intensity, especially for lobster [33], [34]. In addition, inherent differences in site-specific conditions may confound reserve-based assessments because factors such as depth, exposure, and sedimentation rates may help drive differences in the distribution and abundance of lobsters, urchins, and macroalgae between reserves and nearby fished areas [26], [35]. Finally, the process of siting marine reserves tends to select areas of relatively high biodiversity, predator densities, and habitat quality for protection [36], which limit the ability to distinguish between the effects of fishing on community structure versus those caused by other factors. Because much of the ocean's nearshore habitats remain open to fishing, a more thorough understanding of the extent to which fishing triggers trophic cascades is warranted. Identifying the conditions that promote cascades, and determining whether or not they are ubiquitous, may usefully inform the design of marine reserve networks, especially those established to protect kelp forest communities [30].

The California spiny lobster (*Panulirus interruptus*) is the target of one of the oldest commercial fisheries in southern California. Data on commercial landings date back to the early 1900s and have averaged approximately 325 MT in recent years [37]. Spiny lobster populations are considered relatively heavily fished [38], although a recent stock assessment estimates that both total abundance and size structure have stabilized over the last decade [39]. Nevertheless, some believe that over the past century fishing has led to a decrease in overall abundance and individual size of spiny lobsters [38]. Such decreases have the potential to diminish the role of lobsters as effective sea urchin predators. The two main objectives of our study were to: (1) examine the patterns of abundance among lobster, sea urchin (*Stronglyocentrotus* spp.), and macroalgae in southern California giant kelp forests to evaluate whether they are consistent with the hypothesis that lobsters control urchins through predation, and urchins control macroalgae through grazing; and (2) determine whether the biomass of macroalgae was inversely related to the intensity of commercial lobster fishing as predicted by a top-down trophic cascade involving lobsters, sea urchins, and macroalgae. We used a correlative statistical approach to compare the abundance of organisms within three trophic levels, specifically California spiny lobsters (predator), red and purple sea urchins (grazers), and giant kelp and understory macroalgae (primary producers). As such, we did not directly test for the presence of the trophic cascade nor for the impact of fishing on the cascade, which would have required a large-scale, long-term field experiment. However, unlike most studies involving marine reserves, our analyses used sites that were explicitly selected to represent the range of natural variation in the region's kelp forests [19], [40], [41], which were subjected to varying levels of fishing intensity over an eight-year period. The results from our study provide a reasonable assessment of the strengths of the trophic relationships among lobsters, urchins, and macroalgae in southern California's giant kelp forests, as well as the extent to which lobster fishing triggers a top-down trophic cascade. Exploring whether ecological paradigms operate generally across space and time is necessary to advance ecology [16], [42]–[44], especially when conceptual models provide the framework for innovative marine resource management, including marine reserve and other spatial-based approaches [45].

## Materials and Methods

Commercial fishery data on the number of lobsters caught and fishing effort, and ecological data on the abundance of sea urchins and macroalgae were used to address our two objectives. We examined whether patterns of abundance indicated the presence of a trophic cascade by evaluating whether the density of sea urchins was inversely related to lobster abundance, and simultaneously whether the biomass of macroalgae was inversely related to the density of sea urchins. We then evaluated whether lobster fishing influenced the trophic cascade by examining whether: (1) the mean abundance of sea urchins at a site averaged over the eight-year study was positively related to the mean intensity of lobster fishing, and (2) the mean biomass of macroalgae at a site was inversely related to the mean intensity of lobster fishing.

**Figure 1:**
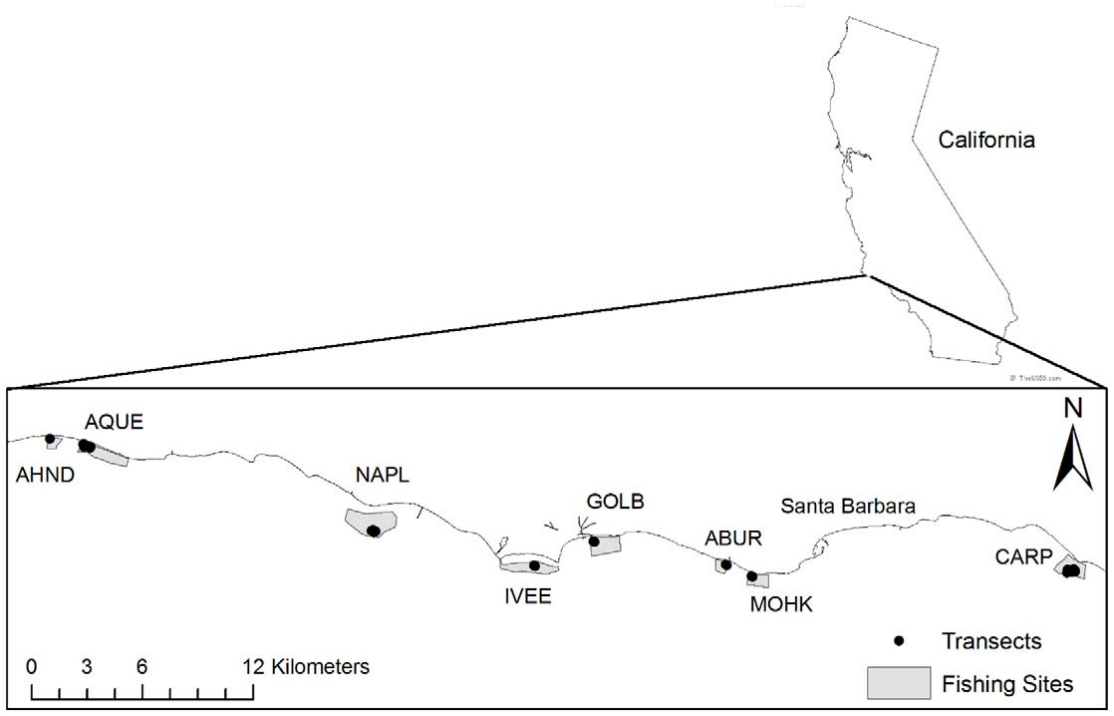
Map of study area along 55 km of Southern California coast. Black dots mark annual transect sites from July 2001 through July 2008 for the Santa Barbara Coastal LTER. Grey polygons mark the 8 trapping areas around LTER sites where commercial lobster fishermen reported daily effort and catch. Mean polygon area was 1.23 km^2^ with the largest being 2.8 km^2^ and the smallest 0.44 km^2^. Abbreviations and numbers of transects sampled at each site are as follows (AHND = Arroyo Hondo, 2 transects; AQUE = Arroyo Quemado, 6 transects; NAPL = Naples, 7 transects; IVEE = Isla Vista, 2 transects; GOLB = Goleta Beach 2 transects; ABUR = Arroyo Burro, 2 transects; MOHK = Mohawk, 2 transects; CARP = Carpinteria, 7 transects).

The data used in our analyses were collected from eight kelp forest sites located within a 50 km stretch along the mainland coast of the Santa Barbara Channel from 2001–2008 (Figure 1). The kelp forest communities at these sites are monitored annually by the Santa Barbara Coastal Long Term Ecological Research (SBC LTER) project and were selected for long-term study to represent the natural range of variability in giant kelp forests in the region [19], [46]. All eight sites were subjected to fishing during the eight-year study period. Oceanographic conditions during this time were generally representative of the region and did not include any major El Niño events [19].

No specific permits were required for fishery data or ecological sampling, or for access to sampling areas. We applied for, and were granted, a Human Subjects exemption from the University of California, Santa Barbara Institutional Review Board (IRB) for our interviews with fishermen. We satisfied all requirements for an exemption and obtained in-person verbal informed consent from all fisherman participants. We documented this by their participation and willingness to proceed with the interview process. All data have only been reported in aggregate and no personally-identifying information is presented.

### Ecological data

Data on the abundance of macroalgae and density of sea urchins were collected along fixed 40 m transects at the eight SBC LTER kelp forest sites in the summers (July–August) of 2001 through 2008 (n = 2 to 7 transects per site at water depths ranging from 4 to 14 m). The number of giant kelp (*Macrocystis pyrifera*) and understory kelp (e.g., *Pterygophora californica* and *Laminaria farlowii*) were counted in a 2 m wide area centered around each 40 m transect and their abundance was estimated as density (number m^−2^). The abundance of low lying understory species of brown, red, and green algae (which are difficult to count as individuals) was estimated as percent cover based on their presence superimposed upon a uniform grid of 80 points placed in a 1 m wide swath centered along each 40 m transect [46]. We converted data of macroalgal density and percent cover to biomass (g dry mass m^−2^) to obtain a single metric for macroalgal abundance for use in our analyses. This was done for giant kelp using the relationship between frond density and biomass derived by Reed et al. [47]. Percent cover and density data for understory species were converted to biomass using the species-specific relationships derived by Harrer [46]. Calcareous species such as upright and encrusting coralline algae were not included in estimates of macroalgal biomass because these species do not form an important part of the diet of sea urchins when non-calcified algae are present [20].

The red and purple sea urchins (*Strongylocentrotus franciscanus* and *S. purpuratus*) are the most abundant sea urchins in kelp forests off California and their extensive grazing on macroaglae and kelp forest community structure has been well documented [48]–[52]. The densities of red and purple urchins were measured in fixed 1-m^2^ quadrats distributed uniformly along each transect (n = 6 quadrats per transect). Purple urchins comprised more than 88% of all urchins counted at the eight sites during the eight-year study period. Two size categories of urchins were recorded, those ≤25 mm in test diameter (which represent individuals <1 year old), and those >25 mm in diameter.

Shears et al. [26] examined trophic cascades associated with fishing and marine reserves on New Zealand rocky reefs, and hypothesized that small-scale topographic complexity of reefs influenced urchin abundance by providing refuge from wave disturbance and predators, including spiny lobsters. We evaluated the effects of small-scale topographic complexity in our study by examining whether urchin density was related to the level of reef rugosity. Rugosity was measured as the length of 1 cm-linked chain required to contour the bottom along a 10 m distance perpendicular to the transect (n = four 10 m distances per transect).

### Lobster fishing data

Spiny lobsters forage actively at night and occupy cryptic habitats during the day rendering daytime visual survey data collected by SBC LTER inadequate for estimating the abundance of lobster. Consequently, we used lobster fishing data to estimate the abundance of legal sized (>83 mm carapace length) lobsters at our study sites. We did not collect data on sub-legal sized lobsters. The commercial and recreational spiny lobster fisheries in southern California have greatly reduced the relative abundance of large lobsters, and most of those caught are considered medium-sized (i.e., relatively close to 83 mm in carapace length) [7].

We worked with fishermen to identify lobster trapping areas that spatially overlapped with the eight kelp forest sites sampled by the SBC LTER (Figure 1). This included overlaying maps of trapping areas obtained from fishermen's interviews with bathymetric data within a Geographic Information System to identify trapping areas (i.e., polygons) within the bounds of the kelp forests that were sampled by the SBC LTER. The specific methods and detailed results of the surveys with fishermen are reported elsewhere [53]. We then summarized catch data derived from logbooks that reported daily fishing effort and catch by trapping area for the eight fishing seasons. Logbook data were provided by the California Department of Fish and Game (CDFG), and permission for their use in this study was granted by individual fishermen. Our total of 2,484 individual trap “samples” (i.e., a trap pulled aboard by fishermen) across all SBC LTER sites accounted for 38% of the total fishing activity along the ∼50 km section of the Santa Barbara Channel's mainland coast spanned by the SBC LTER study reefs.

We calculated the number of lobster caught each year within the bounded trapping area of each site as a proxy for annual lobster abundance at a site, and the cumulative number of traps set within the trapping area of each site in each year as a proxy for annual fishing effort. The number of lobster caught in traps is a useful means of estimating lobster abundance [54]. We constrained our estimate of lobster abundance to the number of legal-sized lobsters because we had no data on the number of sub-legal sized lobster. We think this is reasonable because most of the predation of urchins by lobsters is probably done by legal-sized lobsters [52].

The lobster fishery in southern California is from October to March, yet for most seasons >80% of the annual catch is taken in the first six weeks of the fishing season. As such, we reasoned that number of lobsters caught during each fishing season represented a reasonable estimate of lobster abundance during the summer (i.e., the previous July–August) when data on sea urchins and macroalgae were collected. Based on our interviews with 21 fishermen, we were able to match the reported location of the catch to a polygon that contained the LTER sampling sites. The larger sampling area used to characterize the abundance of lobster at each site (mean polygon area ± SD = 1.23 km^2^±0.79 vs. two to seven 80 m^2^ transects per site for urchins and macroalgae) was needed because lobsters are highly mobile foragers and occur at much lower densities than urchins and macroalage.

Fishing intensity (i.e., trap density) at a site was estimated as the average number of traps deployed and pulled (i.e., “sampled”) per day within each fishing polygon during the first two months of each season. We scaled this to the area (km^−2^) of the fishing polygons, and refer to the metric as trap density. We constrained trap density to the first eight weeks of the season when fishing was most intense and most of the lobsters were caught. We reasoned that average trap density was a good proxy for fishing intensity because the number of lobsters caught per trap [i.e., catch-per-unit-effort (CPUE)] was similar at all eight sites (1-way ANOVA; mean square = 0.09, *F*_7,56_ = 0.312, *P* = 0.946). Rather than varying among sites, CPUE scaled well with the total catch (r^2^ = 0.969), suggesting that fishermen effectively target areas where lobsters are abundant.

### Statistical analysis

To address our question as to whether there was evidence for trophic relationships among lobster, urchins, and macroalgae that were consistent with a top-down trophic cascade, we used a regression approach that implements a three-stage joint iterated Generalized Least Squares (GLS) model. Our approach evaluated the degree to which urchin density was related in time and space to lobsters caught (i.e., our proxy for abundance), and *simultaneously* the degree to which macroalgal biomass was related to urchin density. We used three versions of the model, each of which was fully factorial because it considered the response among the three trophic levels at each site (n = 8) during each time step (n = 8 years).

In Model 1, we used *site* as a fixed effect to account for any unmeasured differences among locations (e.g., exposure to ocean swell, sedimentation, numbers of additional top predators) that may have influenced the relationships among lobster, urchin, and macroalgae. Fixing the effect of *site* accounted for potential underlying differences by subtracting - for each location - the mean of the dependent variable over all sites from the mean value at each site. Model 1 also used *year* (2001–2008) as a fixed effect, thereby accounting for any underlying differences due solely to temporal trends occurring at all locations. Thus, the regression Models 1–3 examined evidence for top-down control of urchins by lobsters, and macroalgae by urchin grazing in all sites and years simultaneously, accounting for correlation between sites and years.

The form of Model 1 was as follows:
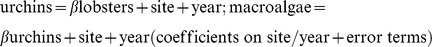
where *urchins* = urchin density (number m^−2^); *lobsters* = lobster catch [number caught km^−2^), site = 8 kelp forest sites, year = 8 years, *macroalgae* = biomass of non-calcareous macroalgae (kg m^−2^), and *β* = the correlation coefficient. The two dependent variables (*urchins* and *macroalgae*) were run simultaneously on independent regressors (urchins were regressed against *lobster* and *macroalgae* were regressed against *urchins*). The simultaneity of this analysis allows correlation across years and sites to be used in estimation, which is needed to evaluate the existence of a trophic cascade in which a change in one trophic level affects other trophic levels. If lobsters indirectly increase macroalgal biomass by consuming sea urchins as predicted by the trophic cascade, then we would expect a significant negative relationship between lobster and urchin abundances, and a significant negative relationship between urchin and macroalgal abundances in our regression model.

Model 2 was similar to Model 1, but instead of fixing site, we used substrate rugosity as a covariate for each site. We ran this model because we hypothesized - based on results of Shears et al. [27] - that the physical complexity of a reef influences urchin abundance by providing them with physical refuge from lobster predation and physical disturbance. Therefore, Model 2 had *site* as part of the random (i.e., pooled) error and *year* as a fixed factor, thus producing a test of the degree to which urchin density was related to lobster abundance, and macroalgal biomass was related to urchin density and substrate rugosity.

The form of Model 2 was as follows:
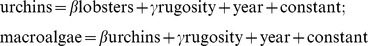
where *urchins* = urchin density (number m^−2^); *lobsters* = lobster catch [number caught km^−2^), *rugosity* = reef substrate complexity, year = 8 years, *macroalgae* = biomass of non-calcareous macroalgae (kg m^−2^), and *β and γ* = the regression coefficients.

Model 3 was similar to Model 2 except that an additional covariate, *water depth*, was added. This model was constructed because water depth can influence the composition of rocky subtidal reefs in many ways, including modulating physical wave disturbance and light availability.

To address the question of whether lobster fishing intensity influences macroalgal biomass by altering the abundances of sea urchins, as predicted by the trophic cascade hypothesis, we compared the relationships between urchin density and lobster fishing intensity (i.e., number of lobster traps deployed and pulled km^−2^ of fishing area that overlapped with the kelp forests/rocky reefs sampled by the SBC LTER), macroalgal biomass and urchin density, and macroalgal biomass and lobster fishing intensity across all eight sites. For each site, we averaged data from all eight years for each of the three variables (lobster fishing intensity, urchin density and macroalgal biomass) and compared the relationships with simple linear regressions. We reasoned that mean trap density averaged across all years was a good indicator of the intensity of fishing at a site and that the time averaged means of urchin density and macroalgal biomass adequately characterized the abundances of primary producers and consumers at each site during the study period. If fishing triggered a trophic cascade, then we predicted that lobster trap density would be positively related to urchin density and inversely related to macroalgal biomass.

## Results

**Figure 2:**
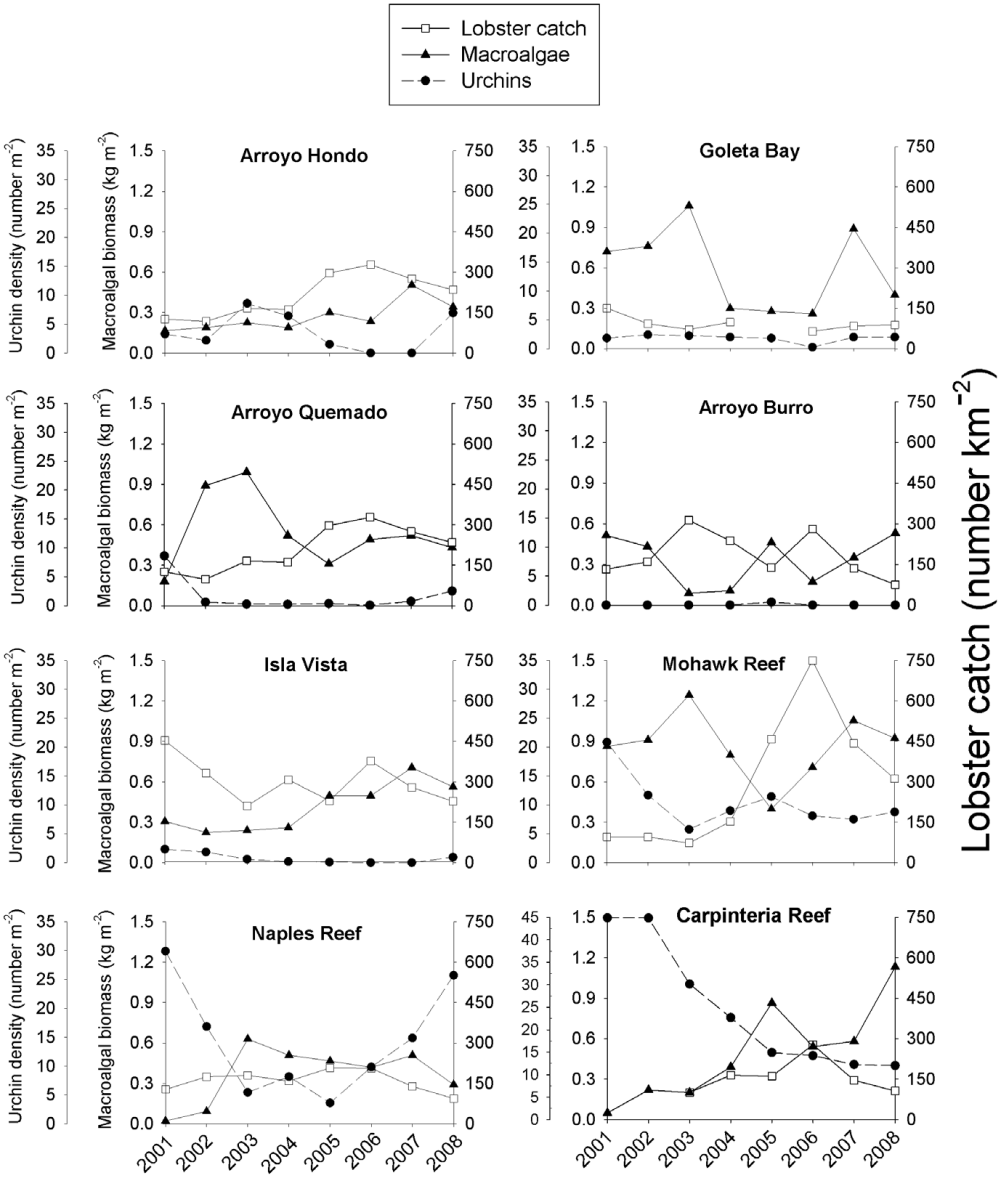
Relationships among lobster catch (a proxy for lobster abundance), urchins, and macroalgae. Lobster catch [legal-sized (≥83 mm carapace length) lobsters only] in number of individuals caught in traps km^−2^, urchin density, and total macroalgal biomass at each of the eight sites from 2001–2008. Macroalgae consisted of non-calcareous species, including giant kelp *Macrocystis pyrifera*. Data for lobster catch were not recorded in 2005 for Goleta Bay, and in 2001 and 2002 for Carpinteria Reef. Note the differences in scale for urchin density at Carpinteria Reef.

Commercial lobster catch, which we used as a proxy for lobster abundance, as well as the density of urchins and biomass of macroalgae varied substantially across the eight study sites during 2001–2008. Urchin density was consistently low (<5 m^−2^) at five sites, Arroyo Hondo, Goleta Bay, Arroyo Quemado, Arroyo Burro, and Isla Vista (Figure 2). It was difficult to detect meaningful relationships visually between lobster and urchin data, except perhaps at Mohawk, Naples, and Carpinteria Reefs, sites that supported relatively high densities of urchins (10–45 m^−2^). The five sites with low urchin abundances had relatively high and stable lobster abundances. Across all sites and years, there were 5 to 24 times more purple urchins (*S. purpuratus*) than red urchins (*S. franciscanus*), and for both species, there were 7 to 73 times more large urchins (>25 mm in diameter) than small urchins (≤25 mm in diameter).

Macroalgal biomass varied independently of urchin density across sites and years except at Naples and Carpinteria Reefs, where large declines in urchin density coincided with increases in macroalgae (Figure 2). Macroalgae consisted primarily of giant kelp and non-calcareous understory algae (e.g., *Pterygophora californica*, *Desmarestia ligulata*, *Chondracanthus* spp., *Rhodymenia* spp.). Giant kelp accounted for 43–99% of the macroalgal biomass in a third of all samples (i.e., individual site-year combinations), 10–40% of macroalgal biomass in half of the samples, and <10% of the biomass in 21% of the samples. Calcareous algae (which were not used in our analyses) comprised <2% of the total algal biomass on average. The biomass of non-calcareous algae (kelp+non-calcareous understory) was unrelated to urchin density when examined over all sites and years (r^2^ = 0.01, *F*_1,63_ = 1.62, *P* = 0.207).

The three-stage least squares regression analysis was designed to detect relationships between the three focal trophic levels graphed in Figure 2. Results of the Model 1 regression indicated that urchin densities did not vary significantly with lobster abundance (Table 1A). However, there was a negative relationship between urchins and lobsters at several of the sites (Figure 2), although not statistically discernable from zero. Model 1 also found no significant relationship between urchin density and macroalgal biomass (Table 1A).

**Table 1:** Three-stage joint least-squares regression.

	Urchin density (no. m^−2^)	Macroalgal biomass (kg m^−2^)
A. Model 1		
Lobster catch	−0.019 (0.031)	
Urchin density		0.028 (0.053)
B. Model 2		
Lobster catch	−0.025 (0.028)	
Urchin density		0.010 (0.015)
Rugosity	242.31 (22.34)**	−1.14 (3.85)
C. Model 3		
Lobster catch	0.001 (0.002)	
Urchin density		0.011 (0.037)
Rugosity	311.17 (49.81)**	−1.21 (16.67)
Depth	−27.77 (22.56)	−0.13 (2.88)

Results of three-stage joint least-squares regression analyses testing the response of urchin density as a function of lobster catch (in number of individuals km^−2^; our proxy for lobster abundance), and the response in macroalgal biomass as a function of urchin density across the eight sites from 2000–2008 (n = 61 observations). Site and year were fixed factors in Model 1. Model 2 had year as a fixed factor, but used reef rugosity as a covariate for each site, instead of fixing site as a factor. Model 3 was the same as Model 2, but included water depth as a covariate at each site. Numbers in the table are regression coefficients. Standard errors of the regression coefficients are shown in parenthesis.

* = *P*<0.05;

** = *P*<0.01.

Results of Model 2 regression (which accounted for variation among years and site specific variation in reef habitat complexity) indicated that much of the variation in urchin density among sites was due to differences in reef rugosity (Table 1B): urchin density increased dramatically with this measure of substrate complexity. Model 2 also found no significant relationship between urchins and macroalgae, and revealed a weak negative relationship between macroalgal biomass and reef rugosity. Model 3, which incorporated water depth as an additional covariate, revealed nearly identical relationships to those observed in Model 2: depth failed to explain any significant among-site variation in urchin density and macroalgal biomass (Table 1C).

**Figure 3:**
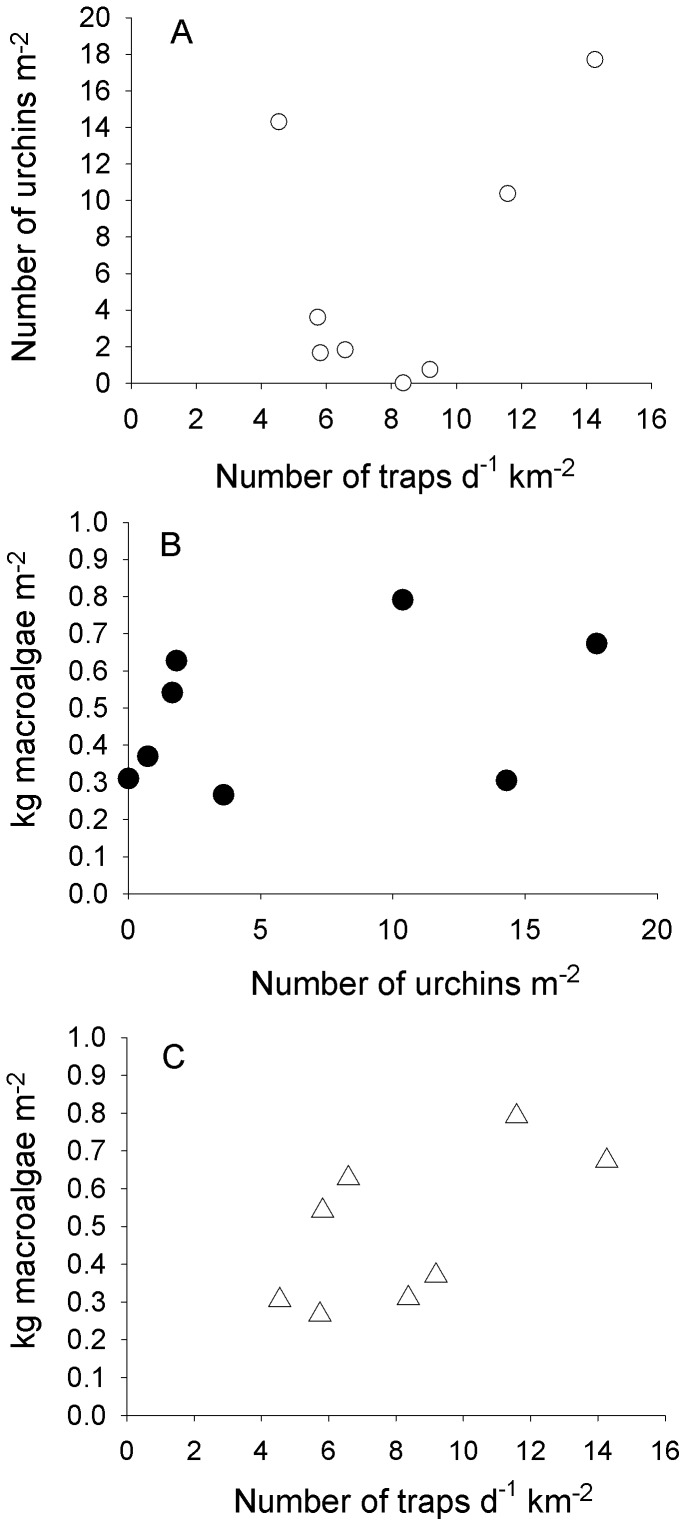
Relationships among trophic levels and lobster fishing intensity. Data represent the mean values for the eight sites shown in Figure 2, each datum pooled across all eight years (2001–2008) (excluding the missing few data described in Figure 2). (A) Urchin density as a function of fishing intensity, measured as the mean daily number of lobster traps pulled (“sampled”) per km^−2^ of each fishing polygon (see Figure 1). (B) Total macroalgal biomass as a function of urchin density. (C) Macroalgal biomass as a function of fishing intensity.

We found little evidence that the intensity of lobster fishing, as measured by fishing effort, induced a trophic cascade leading to low macroalgal biomass. However, results do suggest that lobster fishing released top-down control on urchin abundance. Specifically, the relationship between the daily mean density of traps fished and mean urchin density at each site over the eight year period remained statistically insignificant (Figure 3A; r^2^ = 0.2046, *F*_1,7_ = 1.544, *P* = 0.26), but when Naples Reef, which had high urchin densities but the lowest trap density, was removed from the analysis, the relationship between fishing intensity and urchin density was statistically significant and strongly positive (r^2^ = 0.719, *F*_1,6_ = 12.797, *P* = 0.016). This relationship is consistent with the negative relationship between lobster abundance and urchin density found with the GLS regression, and suggests that fishing may reduce top-down control of urchin populations by lobsters at most of the study sites. Despite higher urchin densities in more heavily fished sites, no evidence emerged linking lobster fishing to declines in macroalgal biomass; indeed, the relationship between lobster fishing intensity and macroalgal biomass remained positive (Figure 2C), although not statistically significant (r^2^ = 0.3866, *F*_1,7_ = 3.782, *P* = 0.100). A positive relationship in this case contradicts a trophic cascade triggered by lobster fishing. Finally, there was no significant relationship between mean urchin density and macroalgal biomass (Fig. 3B; r^2^ = 0.1258, *F*_1,7_ = 0.8632, *P* = 0.389), again implying that urchin grazing did not generally control macroalgal abundance at our study sites during the eight year study period.

## Discussion

Our results suggest that a trophic cascade caused by lobster fishing, in which lobster abundance is reduced leading to increases in urchins and subsequent decreases in macroalgae, is not ubiquitous in the Santa Barbara Channel marine ecosystem. While the density of urchins varied slightly with lobster abundance (as measured by lobsters caught), non-calcareous macroalgae biomass (which included giant kelp) remained largely unrelated to red and purple sea urchin density. Thus, the observed relationship between grazer and primary producer remained inconsistent with that expected in a trophic cascade. Sea urchin grazing was clearly evident at some of our sites but it accounted for relatively little of the observed spatial and temporal variability in macroalgal biomass. Variability in macroalgal biomass has been shown to be independent of urchin grazing in other temperate reef systems as well [55], [56]. Variability between urchin abundance and macroalgal biomass in our data was undoubtedly driven by other unmeasured factors. Reed et al. [19] concluded that physical disturbance from waves was the major factor influencing the biomass of giant kelp, the dominant macroalgal species, at the same sites used in our study. Nutrient limitation and urchin grazing also have important influences on macroalgal abundance under some circumstances, including during ENSO events when nitrogen availability is low, and under conditions of severe urchin grazing, such as those experienced in urchin-dominated “barrens” [47]. What causes the development of urchin barrens in southern California appears to be complex interactions among several factors, including urchin density (as influenced by recruitment, predation, and disease), kelp detritus production, and oceanographic conditions that influence kelp recruitment, growth, and persistence [20], [23], [24].

Our analyses failed to detect strong evidence for the control of urchins by lobsters. However, urchin abundance tended to decline with lobster abundance across many sites (Figure 2), although the relationship was not statistically significant in our regression model. In contrast, urchin density increased across all but one site with increasing fishing intensity (Figure 3). Top-down control of urchins by lobsters has been reported in studies that compared communities inside versus outside marine reserves in New Zealand [52] and the Santa Barbara Channel Islands [23], and from patterns observed in relatively long-term ecological data collected in Maine [5] and southern California [7]. Work in Alaska [29] also indicated that sea otters can control sea urchins. Results of our Models 2 and 3 indicated that if top-down control of urchins by lobster occurred, it was probably a context dependent relationship, a phenomenon first reported by Shears et al. [26]. Specifically, three of our sites, Mohawk, Carpinteria, and Naples Reefs, have topographically complex (or rugose) rock substrata, which is excellent habitat for both urchins [26] and lobsters [56]. We found that urchin density increased by 11±4.3 individuals m^−2^ for every 10-cm increase in rugosity m^−1^ length of substrate. This rather dramatic effect of reef topography implies that predation is probably of relatively minor importance in controlling urchin abundance in habitats with many reef cracks and crevices.

Our results do not include estimates of small, sub-legal lobsters, which may prey preferentially upon small sea urchins. Had we included such data, the addition of small lobsters would have increased the density of lobsters at some sites, likely reducing the negative response of urchins to lobsters. In addition, most of the sites in our study are fished for red sea urchins, which may help explain why there were fewer red than purple urchins. If urchin fishing were not occurring at our sites, the negative relationship between lobsters and urchins may have been weaker as both urchins and lobster prefer reef habitats that provide similar types of shelter. Finally, prior studies that have reported strong top-down control of urchins by lobsters also report that urchin populations often display a bi-modal size structure, with many large and small urchins and relatively few medium-sized individuals, which are preferred by spiny lobster [51]. We found relatively few small urchins at all of our sites, which is not consistent with a bimodal size structure caused by lobster predation. Thus, explanations for the negative relationship between lobster and urchins that we observed should be made with caution, in part because like many ecosystems the Santa Barbara Channel is impacted by multiple anthropogenic disturbances.

Our finding that lobster fishing did not trigger a cascade that reduced macroalgal abundance reflects our observation that both urchin density and macroalgal biomass increased with lobster fishing intensity (Figure 3A,C). This result is consistent with previous findings that urchin grazing is not the primary factor controlling giant kelp biomass at our study sites [19]. A similar result is found in kelp forests where urchins are not important grazers [54], such as in southern Australia where kelp production is heavily influenced by anthropogenic nitrogen inputs [55]. Increases in macroalgae with increased fishing intensity of lobster would be expected if macroalgae were primarily controlled by sea urchins. Our interviews with Santa Barbara Channel fishermen indicated they usually target kelp forests for lobster fishing, which is supported by a quantitative assessment conducted by Guenther [C. Guenther, *unpublished data*] indicating that lobster catch increased with the amount of kelp surface canopy. Lobster trap fishermen also assert that they target areas with consistently high kelp cover [57]. This makes ecological sense if macroalgal biomass is predominantly greater in less disturbed areas because disturbance also negatively impacts lobster populations [53].

Overall, our results found support for the hypothesis that lobsters have top-down control on urchins through predation, a trophic interaction that has been reported previously [5], [9], [13]. However, we found no evidence that lobster fishing indirectly impacts macroalgal populations through increases in the abundance of sea urchins. Instead, our results support the theory that trophic-cascades are context dependent [58], and that although humans have profound impacts on the marine environment through fishing [10], those impacts remain heterogeneous across space and time. Our study highlights an opportunity for long-term ecological monitoring programs to incorporate fishing data where appropriate towards improved understanding of fishing's role in community ecology. Campbell et al. [59] caution ocean managers and conservationists from continuing down the traditional path of treating human behavior as external agents in ecological processes. A better understanding of site-specific processes and identification of the critical variables that make a system resilient or vulnerable to certain activities remains necessary for fostering positive progress in area-based ecosystem management. As resource agencies develop spatial ecosystem-based management we may benefit from enhanced knowledge of when and where human activities most influence ecosystem processes.
